# Fire carbon emissions over maritime southeast Asia in 2015 largest since 1997

**DOI:** 10.1038/srep26886

**Published:** 2016-05-31

**Authors:** V. Huijnen, M. J. Wooster, J. W. Kaiser, D. L. A. Gaveau, J. Flemming, M. Parrington, A. Inness, D. Murdiyarso, B. Main, M. van Weele

**Affiliations:** 1 Royal Netherlands Meteorological Institute, De Bilt, The Netherlands; 2 King’s College London, Department of Geography, London WC2R 2LS, UK; 3 NERC (Natural Environment Research Council) National Centre for Earth Observation (NCEO), Reading, UK; 4 Max Planck Institute for Chemistry, Mainz, Germany; 5 Center for International Forestry Research, Bogor, Indonesia; 6 European Centre for Medium-Range Weather Forecasts (ECMWF), Reading, UK; 7 Department of Geophysics and Meteorology, Bogor Agricultural University, Bogor, Indonesia

## Abstract

In September and October 2015 widespread forest and peatland fires burned over large parts of maritime southeast Asia, most notably Indonesia, releasing large amounts of terrestrially-stored carbon into the atmosphere, primarily in the form of CO_2_, CO and CH_4_. With a mean emission rate of 11.3 Tg CO_2_ per day during Sept-Oct 2015, emissions from these fires exceeded the fossil fuel CO_2_ release rate of the European Union (EU28) (8.9 Tg CO_2_ per day). Although seasonal fires are a frequent occurrence in the human modified landscapes found in Indonesia, the extent of the 2015 fires was greatly inflated by an extended drought period associated with a strong El Niño. We estimate carbon emissions from the 2015 fires to be the largest seen in maritime southeast Asia since those associated with the record breaking El Niño of 1997. Compared to that event, a much better constrained regional total carbon emission estimate can be made for the 2015 fires through the use of present-day satellite observations of the fire’s radiative power output and atmospheric CO concentrations, processed using the modelling and assimilation framework of the Copernicus Atmosphere Monitoring Service (CAMS) and combined with unique *in situ* smoke measurements made on Kalimantan.

In the months of September and October 2015, southern Sumatra and southern Kalimantan experienced increasingly dry conditions until the onset of seasonal rains by the end of October. The suppression of precipitation over large parts of maritime southeast Asia has been found characteristic of strong El Niño events^1^ (Fig. S1). The drought intensified the flammability of a landscape whose susceptibility to fire^2^,^3^ had been increased over the past few decades by seasonal clearing of forest, and by draining of peatlands for agriculture^4^,^5^. Fire is widely used in these regions to clear low, degraded re-growing vegetation and maintain land for the growing of crops. However, during prolonged dry conditions these fires can ignite the desiccated surface of the carbon-rich peat soils, leading to smouldering and even underground burning of the drained peatlands of Sumatra and Kalimantan, far beyond the intended areas. Burning can also spread into relatively undisturbed areas of swamp forest. The smoke particles released from the extensive fires strongly impacted on regional air quality and the health of millions of people^6^. The fires were found mostly impossible to extinguish until persistent rain related to the southward seasonal shift of the Inter Tropical Convergence Zone brought relief by the end of October (Fig. S1).

Robust, quantitative estimates of the carbon emissions from landscape-scale burning are difficult to make^7^. Bottom-up estimates require input parameters such as the area burned and the fuel consumption per unit area^8^, both of which can be associated with high levels of uncertainty^7^. Alternative top-down approaches use atmospheric CO_2_ observations in combination with inverse modelling techniques to estimate CO_2_ fluxes. However, the pyrogenic CO_2_ signal is typically smaller than the natural CO_2_ fluxes associated with vegetation growth and respiration, whilst surface CO_2_ observation networks are sparse, and present-day satellite CO_2_ observations still have limited signal-to-noise ratio and sparse spatio-temporal coverage. Attempts^9^,^10^ at top-down estimation of fire-emitted carbon therefore have mainly relied on observations of carbon monoxide (CO), which offer a much stronger contrast with the undisturbed atmosphere compared to CO_2_, even though the CO emissions from vegetation fires represent a relatively small fraction of the total carbon being released.

To derive the regional CO emissions we use the CAMS modelling and assimilation framework (see also http://atmosphere.copernicus.eu/), which includes assimilation of satellite observations of the fire radiative power (FRP) being emitted by landscape burning. These FRP observations are provided by the Moderate Resolution Imaging Spectroradiometer (MODIS) instruments onboard the Terra and Aqua satellites. The resulting CO emission estimates are optimized through tropospheric composition model simulations constrained with total atmospheric CO column amounts derived from the Measurements of Pollution in the Troposphere (MOPITT) instrument, also operating on Terra. From these constrained regional CO emissions, the total biomass burning carbon emissions are then estimated via a set of unique biomass burning emission factors of CO, CO_2_ and methane (CH_4_) and their respective ratios, derived via unique *in situ* measurements of smoke made downwind of Indonesian landscape fires burning peat and vegetation.

## Results

**Figure 1:**
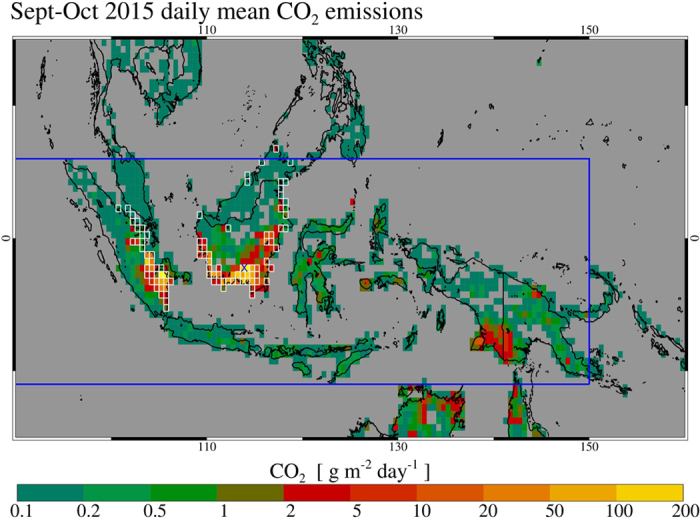
Daily mean CO_2_ emissions from peat and vegetation fires burning across maritime southeast Asia in Sept-Oct 2015, presented in 0.5° × 0.5° grid cells. Cells containing peat soils according to landcover data used in GFAS^23^ are outlined in white (see Supplementary Information B). Locations of our *in situ* trace gas measurements lie close to the Central Kalimantan Capital of Palankaraya, Kalimantan (113.92°E, 2.21°S), indicated with the blue cross (See also Fig. 3). The thick blue line indicates the border of the study domain (east part only shown, full range 70°E–150°E; 11°S–6°N). Map was generated using IDL v8.4 software, http://www.exelisvis.com.

We present first the derived CO_2_ and total carbon emissions over the region as based on the *in-situ* observed emission factor ratios and applied to the CO emissions from the CO budget analysis, both elements of which are explained in detail below. Figure 1 shows the spatial distribution of mean fire-emitted CO_2_ for Sept-Oct 2015, the period over which we calculate that 77% of the total 2015 fire emissions occurred in the region (in 1997 this was 78%)^11^. In addition to CO_2_, significant amounts of CO and CH_4_ were released by these fires, which once oxidized also contribute to the resulting atmospheric CO_2_ concentration increase. The total carbon released by the fires during Sept-Oct 2015 was 227 ± 67 Tg C, of which 83% is in the form of CO_2_ (692 Tg CO_2_), 16% CO (84 Tg CO), and 1% CH_4_ (3.2 Tg CH_4_). The corresponding annual total CO_2_ (and carbon) emissions are estimated as 289 Tg C (884 Tg CO_2_), of which the vast majority (97%) originate from burning in Indonesia, specifically in the south of Kalimantan, the southeastern provinces of Sumatra, and Papua. The associated CO_2_-equivalent emissions, including oxidized CO and contributions from CH_4_ and N_2_O, reach up to 1.2 Pg CO_2_-eq (see Supplementary Information A). A similar trace gas distribution was reported for fire emissions over the same region during Sept-Oct 1997: 86% CO_2_, 12% CO, and 2% CH_4_, respectively^11^. Our total carbon emissions estimate for the 2015 fires represents only a quarter of the most recently reported^11^ estimate of 866 Tg C calculated for the extreme Sept-Oct 1997 fires in the same region, with several earlier estimates showing a large range for this prior event^8^,^9^,^11^,^12^. Nevertheless, the associated mean CO_2_ emission rate of 11.3 Tg day^−1^ for the 2015 fires exceeds the current fossil fuel CO_2_ release rate of the European Union (EU28) (8.9 Tg CO_2_ day^−1^)^13^. The annual fire carbon emissions have led to a maximum contribution of 0.14 ppm (see Supplementary Information A) to the anomalously high global CO_2_ growth rate observed in 2015^14^. Also they have not offset the recently projected^13^ decrease of the anthropogenic CO_2_ growth rate associated with worldwide changes in fossil fuel use. Nevertheless, most of the released carbon from these peat and deforestation fires constitutes a permanent addition of CO_2_ to the atmosphere, because only a fraction will be balanced by vegetation regrowth^13^ and because many are burning ancient peat deposits that have accumulated organic matter over thousands of years^15^ and which constitute amongst the largest global near-surface reserves of terrestrial organic carbon^16^. Similarly, even though the excessively high CO_2_ growth rate noted in 1997–1998 - the largest on record since regular CO_2_ observations by Keeling *et al.* began in the 1950 s - was primarily attributed to reduced net primary production over dry tropical land areas^3^,^17^, part of that atmospheric CO_2_ concentration increase was also driven by the amplified fire emissions^18^.

**Figure 2:**
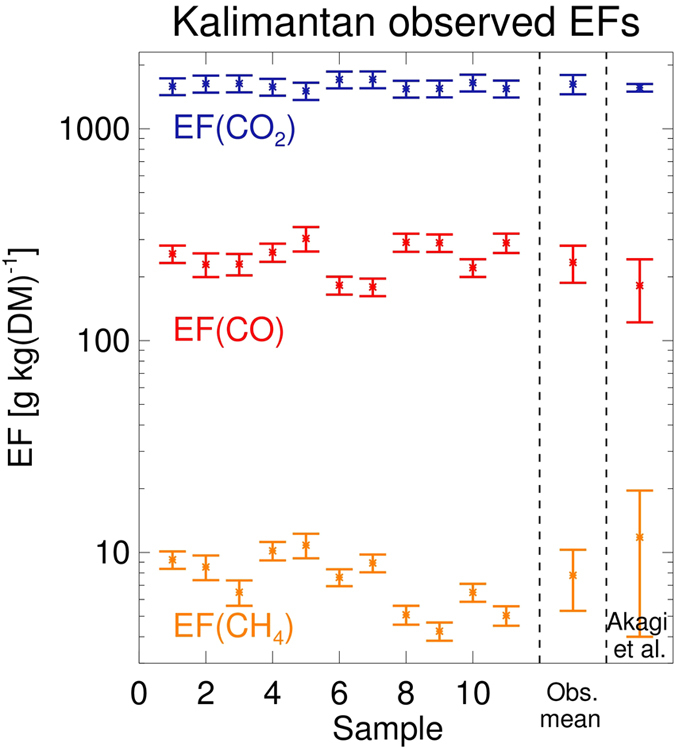
Emission factors (EFs) for CO, CO_2_ and CH_4_ calculated for individual tropical peat fires (g kg^−1 ^DM) determined from *in situ* trace gas measurements made within smoke plumes on Kalimantan, Indonesia. The mean and standard deviation per plume, along with the average over all plumes, are shown along with the EFs contained within the Akagi *et al.*^19^ database. All fires shown here burned on peat soils and were dominated by peat only fuel consumption, except that on 14 October (sample 7, location 3, see Fig. 3) when a smoke plume was sampled that came from an extremely large fire burning tropical forest atop peat soils.

**Figure 3:**
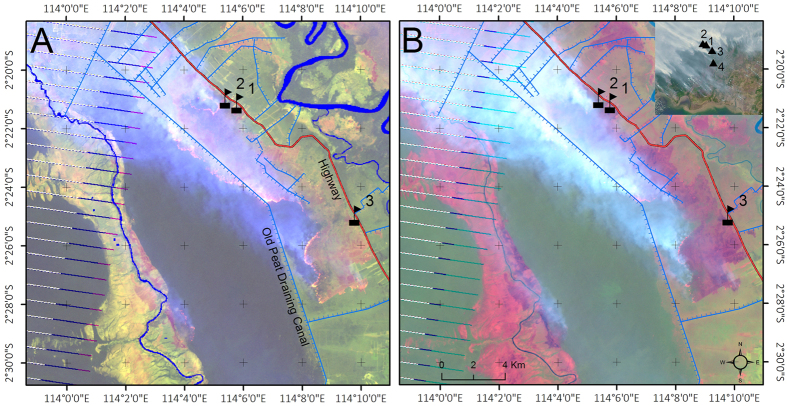
Location of the field sites where smoke for the emissions factor calculations was sampled, approx. 30 km southeast of Palangkaraya, and shown via colour composite satellite imagery from the Landsat Enhanced Thematic Mapper Plus (ETM+) collected on 14 October 09:30 LT at 30 m spatial resolution. (**A**) Data from infrared bands only (RGB = 7,5,3) reveals a 20 km long flaming front progressing across a 16,000 ha peat-swamp forest block. (**B**) Imagery from the visible and infrared bands (RGB = 5,4,3) more clearly shows the thick smoke plume carried northwest by the prevailing easterly trade winds. Image striping is caused by the 2003 failure of the ETM+ Scan Line Corrector (SLC), but do not affect the interpretations made here. Inset in (**B**) shows coarser spatial resolution MODIS colour composite imagery of the same day, indicating the broader scale situation and the presence of large smoke plumes from the burning vegetation and peat. The southern coast of Kalimantan can be seen at the bottom of the inset. *In situ* measurement of these plumes was conducted at the four locations indicated by the flags in the inset, three of which are seen in the ETM+ subscene. On 12 October plumes 1–4 (Fig. 2) were measured at location 1. On 14 October (date of this imagery) plumes 5, 6 (location 2) and 7 (location 3) were measured. On 16 October plumes 8–11 (location 4) were measured. Map created using ArcMap v10.2.2 geospatial processing program http://www.esri.com. The LANDSAT imagery was downloaded from the US Geological Survey website at: http://earthexplorer.usgs.gov/.

The emission factors of CO_2_, CH_4_ and CO applied here for tropical peat burning are shown in Fig. 2. These are derived from *in situ* CO, CO_2_ and CH_4_ trace gas concentration measurements made in smoke at four different locations near Palankaraya, at the centre of the fire-affected zone in the south of Kalimantan (Fig. 3) during 12–16 Oct. 2015. From the measurements made within individual peat fire smoke plumes we calculated mean peatland landscape fire emission factors (EFs) of 234 ± 47, 1625 ± 170 and 7.8 ± 2.5 grams of gas emitted per kg Dry Matter burned (g kg^−1^ DM) for CO, CO_2_ and CH_4_, respectively. For this we applied a best estimate^8^ of 27% for the contribution to the fire-emitted smoke of above-ground vegetation burning, relative to purely peat burning, as per ref. 19. To our knowledge these are the first EFs derived for Indonesian peat and landscape fires via these type of onsite measurements of fire emitted smoke. At our field measurement locations in Kalimantan we saw little evidence of flaming combustion and measured primarily smouldering peat, though a few smoke measurements involved occasional contributions from small clumps of ignited dry vegetation. Our mean EFs for these essentially ‘peat-only’ fires are 255 ± 39 g kg^−1^ DM, 1594 ± 61 g kg^−1^ DM and 7.4 ± 2.3 g kg^−1^ DM, for CO, CO_2_ and CH_4_, respectively, see methods. Prior tropical peat EFs are somewhat different^20^,^21^, being based on combustion chamber investigations and forming the basis for the peat EF description in the widely used Akagi *et al.* compilation^19^. The uncertainties in the derived EFs are mainly driven by an estimated 10% uncertainty in the carbon content of the burning peat (~55% for peat-only fires). On one occasion (14 October, location 3 on Fig. 3) we did measure smoke from what Landsat satellite imagery showed was a fire involving a large flaming fire front of around 20 km length, sustained by substantial vegetation burning atop of peat. Smoke from this fire had EFs of 179 ± 17, 1710 ± 156 and 8.9 ± 0.9 g kg^−1^ DM for CO, CO_2_ and CH_4_ respectively, which in comparison to the smouldering ‘peat only’ fires showed a reduced (increased) EF for CO (CO_2_) due to the substantial contribution from above-ground flaming vegetation. However, over the total lifetime of fires involving peat combustion, the amount of peat consumed typically greatly exceeds that of any above ground vegetation, mainly because dry peat deposits can continue to burn for many days into the ground at a single location whilst also spreading laterally to ignite new peat areas. We use the EFs from measurement location 3, weighted by 27%^8^, in combination with the mean of the peat-only plume samples to compute the mean landscape scale peatland fire EFs reported above.

To derive the total CO_2_ and CH_4_ fire emissions, the ratio of the EFs of CO_2_ and CH_4_ relative to the EF of CO are multiplied by the total regional CO emissions, see below and in Table 1. The observed EF ratios are 7.3 ± 1.6, and 0.035 ± 0.011 for CO_2_/CO and CH_4_/CO, respectively. These are lower by 15% for CO_2_ and by 46% for CH_4_ compared to the Akagi *et al.* compilation^19^, mainly because the EF for CO that we observed during our fieldwork in Kalimantan is higher by 29%. Table 1 provides the full range for the observed EF ratios of CO_2_/CO and CH_4_/CO based on the 11 individually sampled plumes. The quoted uncertainties are based on the spread of the samples, and further on an assumed range of 0–50% (instead of fixed percentage of 27%) in the contribution of above ground vegetation burning to the total fuel consumption, which mainly increases the uncertainty in peatland EF for CO.

To derive the total regional CO emissions we made use of the Composition-Integrated Forecasting System (C-IFS)^22^ for atmospheric composition, which includes biomass burning emissions provided by the Global Fire Assimilation System (GFASv1.2)^23^. GFASv1.2 fire emission estimates are based on daily global MODIS observations of fire radiative power (FRP), which have a ~1 km spatial resolution. The standard GFAS system converts the MODIS FRP to biomass combustion rate^24^, including eight land cover specific factors influenced by a calibration against GFED3.1^25^, and subsequently calculates emission fluxes for a variety of smoke constituents^23^ (see Supplementary Information B). In addition, we use CO observations from the MOPITT satellite instrument to constrain the atmospheric CO abundance over southeast Asia (Supplementary Information C).

**Figure 4:**
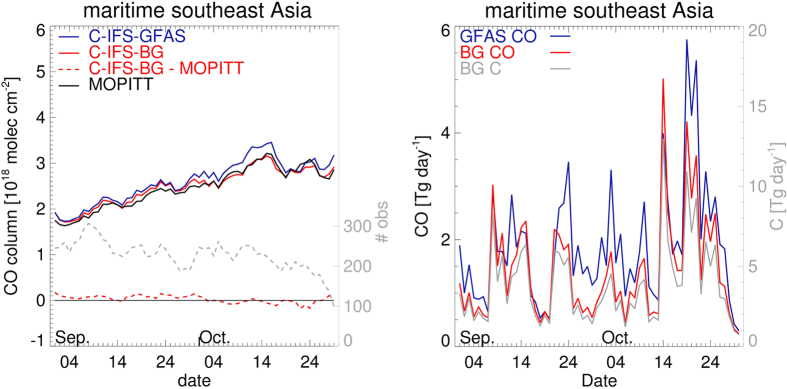
Left panel: Evolution of the 3-day running mean CO column amounts calculated over the maritime southeast Asian region from C-IFS-GFAS (blue) and C-IFS-BG (red, solid), as compared to MOPITT observations (black). Bias of C-IFS-BG CO column amounts with respect to MOPITT are also shown (red, dash). In grey dashes we shown the number of 1° × 1° gridded observational samples (right axis). Right panel: Evolution of GFAS^23^ (blue) and BG (red) CO emissions. In grey the corresponding total carbon emissions (right axis).

As shown in Fig. 4, the temporal evolution of CO as observed by MOPITT is well captured by C-IFS GFAS, though with a small systematic positive bias in the simulations over the maritime southeast Asian region. Over south Kalimantan the simulation shows a negative bias against MOPITT (Fig. S2), which is consistent with our observed *in situ* EF for CO from peat fires measured in Kalimantan being higher than that assumed in GFAS. Also, GFAS may tend to underestimate fire activity due to some erroneous identification by MODIS of extremely thick smoke as cloud, which then masks out fires underneath (Fig. S3). This could prevent identification of some fires in the operationally generated FRP product, and therefore underestimated emissions would have been applied in the C-IFS GFAS simulation. We used the MOPITT CO column observations over the source region to up- or down-scale the GFAS-generated CO emissions, see Methods. Additionally we evaluated the C-IFS model over the full maritime southeast Asian region, i.e. including the outflow regions where reported observational uncertainties^26^ are applicable for C-IFS evaluations with MOPITT (see Methods). The high bias in the outflow region west of Indonesia, but not over Kalimantan (Fig. S2), led us to significantly downscale CO fire emissions over Sumatra. The large geographical extent of extreme aerosol amounts over Sumatra, as recorded by the CAMS operational aerosol analyses (Fig. S3), also points to potential uncertainties in the land cover data used in GFAS that influence aerosol emissions as well as CO emissions. Uncertainty in GFAS land cover is accounted for through the CO emission optimization procedure, as well as through a 5% uncertainty contribution in the conversion to CO_2_ fire emissions (Supplementary Information B). The optimized model simulation resulting from our “best guess (BG)” of fire CO emissions (termed C-IFS-BG) then shows good agreement with respect to the evolution of the MOPITT observed CO over the maritime southeast Asian region, Fig. 4, and Fig. S4 for sub-regions. The right-hand panel of Fig. 4 (Fig. S5 for sub-regions) shows the corresponding daily CO and total C emissions for the peat and tropical forest fires combined. From the C-IFS-BG simulation we derive a total regional CO emissions estimate for Sept-Oct 2015 of 84 ±18 Tg. Applying our *in situ* EF ratios (Table 1) to the optimized CO emissions for peatland fires, while keeping the native GFASv1.2 emission factors for areas of tropical forest (non-peatland) burning, we derive total emissions of 692 ± 213 Tg CO_2_ and 3.2 ± 1.2 Tg CH_4_. Total carbon emissions are then computed from the sum of the carbonaceous components of the individual CO, CO_2_ and CH_4_ emissions, whilst the uncertainty of 30%, corresponding to 67 Tg C, is the square root carbon weighted sum of the relative uncertainties in the CO, CO_2_ and CH_4_ emissions. The uncertainty of 31% (38%) in the CO_2_ (CH_4_) emissions is the square root sum of the relative uncertainty in CO emissions, that of the respective emission factor ratios and the land cover uncertainty, Table 1.

## Discussion

The consistently small daily mean bias in Fig. 4 shows the very large extent to which the C-IFS-BG model simulation is able to appropriately track the MOPITT CO observations from day to day. Application of the C-IFS model optimization to the three sub-regions (Fig. S4) revealed increasing model uncertainty, indicating that in our approach the smallest total uncertainty is obtained by constraining CO emissions in the larger domain including the outflow regions. For the C-IFS-BG model, the uncertainty of 11% as derived for the larger domain, is dominated by the uncertainty in the chemical loss of CO. This uncertainty increases for the sub-regions because of higher relative uncertainties in the chemical loss close to the emission sources^12^. To provide sub-regional constraints on the CO emissions a formal CO inversion^10^ would be advantageous, even though such a study would need to include a careful assessment of the uncertainties in the MOPITT CO observations over the source region, including cross validation against independent (satellite) CO observations such as from IASI^27^.

Our fire carbon emission estimate for Sept-Oct 2015 represents the largest seen over the Maritime southeast Asia region since 1997, but still it is only a quarter of the most recent estimate^11^ for the Sept-Oct period of that El Niño year. We note that fire carbon emission estimates for 1997 are intrinsically more uncertain^8^,^9^,^11^,^12^ than our 2015 estimate, considering that neither MODIS nor MOPITT satellite observations were available before 2000. The more limited carbon emissions for 2015 are most likely associated to the combined effects of reduced burnt area and reduced burn depth. Based on GFEDv4 burned area^11^, the Sept-Oct estimated area of peatland burned is 0.8 × 10^6 ^ha (1997: 1.7 × 10^6 ^ha), implying an average loss of 42 kg DM m^−2^ in peatland (1997: 78 kg DM m^−2^), equivalent to an average burn depth of 26 cm (1997: 50 cm), see Supplementary Information D. Since many areas that burned in 2015 are known to have burned during prior El Niño’s, this indicates the possibility that peatland areas previously burned consumed on average less fuel per unit area. Fire mitigation and control measures may also have been somewhat more effective than in 1997 and also meteorological factors affecting fire extent, such as the level of precipitation preceding Sept-Oct^5^ and the normal onset of the monsoon by the end of October, were not as severe in 2015 as they were during the more prolonged drought of 1997 (Fig. S1).

## Methods

### Field measurements

CO_2_, CO, and CH_4_ emission factors (EFs, in grams released per kg of dry biomass burned) were derived from trace gas mixing ratios measured in smoke plumes using a ground-based, portable cavity enhanced laser absorption spectrometer^28^,^29^, with the measurement capability extended to include CO. Precision (Allan variance, 1 sigma @ 1 Hz) of the mixing ratios was 0.14 ppm for CO, 1.71 ppb for CH_4_, and 2.63 ppm for CO_2_, with a total absolute uncertainty of around 1% of the measured concentrations. Measurements at 1 Hz were made downwind from fires at four sites between 12 and 16 October 2015, with each site located on peat and within ~30 km of Palankaraya (2.21°S, 113.92°E), the capital of Central Kalimantan and one of the most fire affected regions of Maritime southeast Asia during the 2015 El Niño related drought. Whilst most measurements were made in smoke plumes of peat-only fires, one sample derives from a plume coming from extensive burning of tropical forest atop of burning peat (Fig. 3). The smoke from individual fires found at each measurement site was sampled for between 5 to 30 minutes depending upon conditions, and the emission ratio (ER) of each species with respect to CO_2_ derived from the slope of the linear best fit to the relevant trace gas mixing ratios^30^. The gas exchange time of the internal 315 cc cell of the spectrometer was 6 sec, and so the 1 Hz data were subsampled every 10 sec prior to the analysis. The EF for each species was calculated from the ERs using the carbon mass balance approach^31^,^32^, and the strength of the linear best fits used to derive the ERs meant that the EF uncertainty was dominated by the assumed ±10% uncertainty in the fuel carbon content^33^.

### Use of C-IFS to constrain CO fire emissions

Our optimized “Best Guess (BG)” CO emissions tabulated in Table 1 are based on a sensitivity analysis with the atmospheric chemistry transport model C-IFS^22^. Simulations have been evaluated with respect to CO data from MOPITT-V5 TIR^34^. The CO emission estimate with respect to MOPITT has been optimized for both the emission source regions and the outflow region.

First, at the local, 1° × 1° grid box scale, the biases between MOPITT and the C-IFS model simulation of CO over the emission source regions provide information on potential biases in the GFAS CO emissions. We computed the local, instantaneous scaling factors relative to the GFAS emissions, based on the relative enhancements of both MOPITT and the C-IFS model simulation with respect to the climatological CO columns derived from the CAMS Interim Reanalysis (see Supplementary Information C). To account for the measurement uncertainty in individual MOPITT observations over the emission source regions, we averaged the available observations within a 3° × 3° area around each 1° × 1° grid box. On days with missing MOPITT data in the 3° × 3° area, we used the last available ratio until a new observation became available in the area, up to 5 days ahead. Sensitivity experiments using a 5° × 5° area, or with a shorter period in which scaling factors are maintained, provided no significant changes to the CO emission scaling factors.

This local scaling of the CO emissions remains to some extent hampered by the limited amount of MOPITT data available over the fire emission source regions, caused primarily by a larger than normal CO retrieval uncertainty due to the significant presence of smoke aerosols coming from the fires (see Figs S3 and S6). The C-IFS CO field over the emission regions is also associated with relatively large uncertainties, related to the diurnal variation in emissions and the emission injection height into the atmosphere. The local emissions scaling also does not yet account for CO long-range transport and CO lifetime. We used the MOPITT-scaled CO emissions as our first guess for a second optimization of C-IFS against MOPITT, but now also including the outflow region. Away from the fires themselves, the emitted CO has been uplifted through convection, and mean aerosol optical depths are reduced, resulting in a much greater number of MOPITT observations becoming available for use. Together with the better sensitivity of MOPITT to CO in the free troposphere rather than in the boundary layer, this increased number of observations provides a much better constraint on the regional CO burden.

From the evaluation of C-IFS-GFAS against MOPITT over the larger domain spanning from 70°E–150°E and 11°S–6°N, we found a high bias in the outflow region west of Indonesia, but not over Kalimantan and Papua (Figs S3 and S4). This led us to significantly downscale the CO emissions over Sumatra, which we believe is likely associated with uncertainties in GFAS regarding the land cover type specification^35^. This adjustment significantly improved the agreement of the C-IFS-BG CO with MOPITT over the larger domain.

## Additional Information

**How to cite this article**: Huijnen, V. *et al.* Fire carbon emissions over maritime southeast Asia in 2015 largest since 1997. *Sci. Rep.* **6**, 26886; doi: 10.1038/srep26886 (2016).

## Supplementary Material

Supplementary Information

## Figures and Tables

**Table 1:** Emission factor (EF) ratios of CO_2_ and CH_4_ to CO, along with CO total columns, CO, CO_2_, CH_4_ and total carbon emissions, together with their corresponding uncertainties.

	Unit	Mean	RMSE	Rel. uncertainty
EF(CO_2_)/EF(CO)^1^	g kg^−1^ CO_2_/g kg^−1^ CO	7.3 (5.0–9.5)	1.6	22%
EF(CH_4_)/EF(CO)^1^	g kg^−1^ CH_4_/g kg^−1^ CO	0.035 (0.015–0.050)	0.011	32%
MOPITT column mean^2^	10^18^ molec cm^−2^	2.5	0.15	6%
C-IFS-BG column mean^3^	10^18^ molec cm^−2^	2.5	0.3	11%
C-IFS-BG column bias^4^	10^18^ molec cm^−2^	0.03	0.12	17%
CO emissions^5^	Tg CO	84	18	21%
CO_2_ emissions^6^	Tg CO_2_	692	213	31%
CH_4_ emissions^6^	Tg CH_4_	3.2	1.2	38%
Total carbon emissions^6^	Tg C	227	67	30%

All numbers are Sept-Oct 2015 means over the maritime southeast Asian region. RMSE is the root mean square error.

^1^The CO_2_/CO and CH_4_/CO EF ratios for peatlands are based on our *in situ* measurements, with the range in the individual measurement samples given in brackets. The estimated uncertainties are based on the standard deviations of the observed ratios, and the uncertainty in the fraction of above ground vegetation consumed with respect to the total fuel consumption.

^2^MOPITT mean total column CO observations. RMSE refers to the standard deviation of the error of individual column observations^26^.

^3^C-IFS-BG mean total column CO. For the uncertainty estimate see Supplementary Information C.

^4^C-IFS-BG mean bias with respect to MOPITT, and corresponding RMSE of model CO, see Supplementary Information C.

^5^Time integrated CO emissions derived from C-IFS-BG. The relative uncertainty is computed as the square root sum of the relative uncertainties of the model bias with respect to MOPITT, the MOPITT observational uncertainty, and the C-IFS-BG model uncertainty.

^6^Time integrated derived CO_2_, CH_4_ and total carbon emissions, using the observed EF ratios for peatlands (potentially including vegetation atop) and the GFAS native EF ratios for tropical forest burning. The total uncertainty for each carbon component is computed as the square root sum of the uncertainty of the CO emissions, that of the respective EF ratios, and the uncertainty due to the land cover map (5% for CO_2_). Total uncertainty for the total carbon emissions is computed as the weighted sum of the relative contributions from the CO, CO_2_ and CH_4_ emissions.
