# Supplementary material for: Fire carbon emissions over maritime southeast Asia in 2015 largest since 1997
Source: Sci Rep. 2016 May 31;6:26886. doi: 10.1038/srep26886 (PMC4886261; doi:10.1038/srep26886)
Supplement: Supplementary Information [file srep26886-s1.pdf]

Supplementary Information to:

Fire carbon emissions over maritime southeast Asia in 2015 largest since 1997

V. Huijnen[1], M. J. Wooster[2,3], J. W. Kaiser[4], D.L.A. Gaveau[5], J. Flemming[6], M. Parrington[6], A. Inness[6], D. Murdiyarso[5], B. Main[2], M. van Weele[1]

[1] Royal Netherlands Meteorological Institute, De Bilt, The Netherlands.
[2]King's College London, Department of Geography, London, UK
[3]NERC National Centre for Earth Observation (NCEO), Reading, UK
[4]Max Planck Institute for Chemistry, Mainz, Germany
[5]Center for International Forestry Research, Bogor, Indonesia
[6]European Centre for Medium-Range Weather Forecasts (ECMWF), Reading, UK

Supplementary Information A.

The derived annual total fire carbon emissions of ~289 Tg C lead to an injected mass into the atmosphere of 289×44/12 = 1.06 Pg CO_2, corresponding to a CO_2 global growth rate enhancement of 0.14 ppm yr^{-1}. Note that the present-day atmospheric CO_2 volume fraction of about 400 ppm corresponds with a mass of about 3.1 Pg CO_2 (7.81 Pg ppm^{-1}), given the total mass of the atmosphere of 5.148×10^{18} kg. Thus, the annual total 2015 El Niño related fires over maritime southeast Asia are estimated to have contributed to a maximum atmospheric CO_2 accumulation of 0.14 ppm over 2015, of which some small part will have been taken up by the biosphere and oceans. The CO_2 airborne fraction (defined as the ratio of the annual increase in atmospheric CO_2 abundance to the actual CO_2 emissions from anthropogenic sources) is significantly less than one (Ciais et al., 2013), and so the long-term global mean atmospheric CO_2 enhancement related to the permanent conversion of peat carbon will be less than 0.1 ppm.

For the reported annual total CO_2-equivalent emissions of 1.2 Pg CO_2-eq we add the contributions from CO_2 (884 Tg yr^{-1}), CO (104 Tg yr^{-1}), CH_4 (4.1 Tg yr^{-1}) and N_2O (0.14 Tg yr^{-1}) emissions, where GFAS' standard EFs for N_2O of 0.24 g kg^{-1}DM are applied in the absence of *in situ* derived N_2O EFs. IPCC-AR5 Global Warming Potential (GWP) conversion rates of 34 (CH_4) and 298 (N_2O) are adopted to compute CO_2-equivalent emissions.

Supplementary Information B.

The MODIS instruments aboard NASA's Terra and Aqua satellites observe landscape fires with a 1 km spatial resolution at nadir in wavebands from the visible to the thermal infrared. The satellites are in polar orbits with daytime local equator crossing times of 10:30 am and 1:30 pm respectively. The MODIS swath of 2300 km enables equatorial regions to be observed twice during daytime and twice during nighttime on most days, and the standard MODIS fire products (MOD14/MYD14) enable the detection of actively burning fires covering down to around 10^{-4} to 10^{-3} of a pixel (~ 100 to 1000 m^2) based on their strong middle infrared thermal signals, and the calculation of their fire radiative power (FRP) which is proportional to their fuel consumption rate (Wooster et al., 2005). However, clouds can mask the thermal signal of fires from view, and fires are therefore only able to be detected where the pixel is deemed to be cloud-free (Giglio et al, 2003). Smoke from fires is largely transparent to the mid-infrared radiation used as the primary fire measurement waveband, mainly because these aerosols tend to be very much smaller in diameter than middle infrared wavelengths, but the cloud-masking algorithm used in the MODIS fire products has been shown to occasionally interpret particularly thick smoke as cloud, preventing identification of certain fire pixels and thus leading to some underestimation of FRP (Schroeder et al. 2014).

GFAS (Global Fire Assimilation System, Kaiser et al. 2009, 2012) is an operational service in CAMS that provides daily global fire emission estimates in real-time from FRP observations. The data are freely available like other CAMS services. The GFASv1.2 system processes the MODIS fire products MOD14/MYD14 to derive a gap-filled global FRP estimate at 0.1° spatial resolution, converts it to biomass combustion rate, and subsequently calculates emission fluxes for 41 smoke constituents (gases and aerosols). FRP has been shown to be directly proportional to combustion and aerosol emission rates (Wooster et al., 2005, Ichoku and Kaufman, 2005). Heil et al. (2010) showed that at regional scales when derived from MODIS this proportionality varies between biomes, and developed conversions of FRP to biomass combustion rate for use in GFAS across eight different fire types, based on a 0.5° resolution calibration against the well-established GFED3.1 inventory (van der Werf et al. 2010). Vast areas in the East of Sumatra as well as the southern region of Kalimantan have been identified as dominated by peat fires (as indicated in Fig. 1) during this calibration. During carbon emissions calculations for the 1997 El Niño related fires, Page et al. (2002) assumed that the fraction of the total fuel consumption that was comprised of above-ground vegetation was 27% when burns occurred in peatlands, and though uncertain the selection of this fraction effectively limits the attribution to peat with respect to vegetation burning in such areas. Certain peat fires can be more difficult to quantify from space, because the parts burning below ground strongly affect neither the surface emitted thermal radiation nor the burnt area. However, peat fires are generally associated with some vegetation burning above ground since the peat is usually first ignited by burning vegetation that transfers the combustion zone into the soil. In this study, we use GFASv1.2 as a priori estimate for the CO emission rate, and, after C-IFS optimization against MOPITT, apply the *in situ* (locally measured) relative smoke emission factors to calculate the CO_2 and CH_4 emission rates from our C-IFS BG CO emission rates. For fires that are observed outside peatland areas, the native GFAS emission factors for CO, CO_2 and CH_4 for tropical forest vegetation are assumed, which are within the range provided by Akagi et al. (2011).

The uncertainty in land cover speciation is taken into account by the optimization procedure involving MOPITT CO observations, which covers implicitly the uncertainty brought by the conversion from FRP to dry matter burned which is land cover dependent (see Kaiser et al., 2012). In addition, for the conversion to CO_2 emissions a maximum 5% uncertainty remain due to fires in different biomes, considering the different EF(CO_2)/EF(CO) ratios. Given the 25% downscaling of the CO emissions in the optimization procedure, and the large sensitivity of peat area burning to CO emissions illustrated by the different FRP to CO conversion factors for peat compared to tropical forest (Kaiser et al., 2012), the integral land-cover uncertainty is less than 5%, and given the EF(CO_2)/EF(CO) ratios for the different biomes also less than 5% for the CO_2 emissions.

Supplementary Information C.

MOPITT (Measurements of Pollution in the Troposphere) V5 CO column observations (Worden et al., 2010; Deeter et al., 2012) are used to constrain the C-IFS simulated CO total columns. The MOPITT instrument is a multi-channel Thermal InfraRed (TIR) and Near InfraRed (NIR) instrument operating onboard NASA's sun-synchronous polar orbiting Terra satellite. MOPITT provides data with a horizontal spatial resolution of 22 km × 22 km and a swath width of 640 km, providing global coverage every 3 days. MOPITT retrievals of the CO volume mixing ratio profile are derived using optimal estimation (Deeter et al., 2012). The total column CO product is based on the integral of the retrieved CO volume mixing ratio profile. A monthly MOZART-4 (Emmons et al., 2010) climatology serves as the basis of the MOPITT *a priori* profiles. For our study we use the TIR-derived CO total column observations, which are provided both over the oceans and over land. Highest CO sensitivities of these MOPITT TIR measurements are in the middle troposphere, around 500 hPa. Sensitivity to the lower troposphere depends on the thermal contrast between the land and lower atmosphere, which is higher during the day than in the night. Therefore, in our study we only use daytime MOPITT TIR observations. Standard deviation of the error in the MOPITT V5-TIR product is reported as 0.15×10^{18} molec cm^{-2} (Deeter et al., 2013). Daily mean model CO columns have been gridded to a 1° x 1 ° spatial resolution, and for our analysis we applied the MOPITT averaging kernels to the logarithm of the mixing ratio profiles, following Deeter et al. (2012).

Uncertainties in our estimate of the CO emissions is assessed using the CAMS system for forecasts and analyses of atmospheric composition, based on C-IFS (Flemming et al., 2015, Inness et al., 2015a). Tropospheric chemistry in the C-IFS is modelled according to a modified version of the Carbon Bond version 5 (CB05, Flemming et al., 2015). Apart from the fire emissions from GFAS, MACCity anthropogenic emissions and POET (Precursors of Ozone and their Effects in the Troposphere) natural and biogenic emissions (Granier et al., 2011; Olivier et al., 2003) were used for the majority of the remaining emissions components. For isoprene emissions, a 10-year climatology based on the MEGAN-MACC inventory was used (Sindelarova et al. 2014). C-IFS was run on T255 horizontal resolution (corresponding to ~0.7×0.7°) with 60 vertical levels.

The total column C-IFS-BG model simulation uncertainty of 11% as reported in Table 1 was computed as the square root sum of three contributions. Most important is a upper limit of 20% uncertainty in the model CO lifetime, due to the combined chemical effects of the consumption of OH radicals by CO, the

heterogeneous loss of HO_x on aerosols, and the reduction of UV light by aerosols over the tropical Indian Ocean (Duncan et al., 2003). Using C-IFS-BG we calculated a total CO chemical loss over the maritime southeast Asian region of 37 Tg CO. An uncertainty of 20% (7.4 Tg CO) corresponds with 9% relative uncertainty in the total CO emissions (84 Tg CO, Table 1).

Further model uncertainties in C-IFS relate to uncertainties in biogenic emissions and/or the isoprene chemistry scheme (e.g. Fisher et al. 2015). Specifically, Flemming et al. (2015) found that C-IFS has a general positive bias in the tropical burden of CO with respect to MOPITT. We used the CAMS Interim Reanalysis to quantify the local, daily climatological bias in the CO total column in the 2015 model run. This reanalysis covered the years 2003-2014, where MOPITT-V5T CO total columns and a combination of ozone measuring satellite instruments were assimilated according to Inness et al. (2015a). The C-IFS model setup used was very similar to the set-up used in this study, although the reanalysis was run on a somewhat coarser horizontal resolution of T159 (~ 1.1° × 1.1°). Also a control experiment was made where data assimilation was switched off.

For the September-October period, the root-mean-square (RMS) of the reanalysis CO total columns with respect to MOPITT over maritime southeast Asia was computed as 0.02×10^{18} molec cm^{-2}. As MOPITT CO was also assimilated, this is not an independent validation of the CO total columns, but rather a consistency test. This is relevant here as we use MOPITT CO for our CO regional emission estimate. By scaling the RMS with the reanalysis mean Sept-Oct CO total column for the 2003-2014 period (~1.8×10^{18} molec cm^{-2}), we estimate that use of the reanalysis as our reference climatology adds 1% to the total C-IFS model uncertainty, which is the second contribution to the 11% total model uncertainty.

For the computation of the model bias we exclude the year 2006 from the reanalysis, since this is the year of the last relatively strong El Niño. In this way we minimize the contribution in CO model bias due to the fire emissions estimated via GFAS. The climatological model bias is in the range of $0.1\text{-}0.5 \times 10^{18}$ molec cm^{-2}, depending on time and location, with the maximum bias over Sumatra during October, see Fig. ED6. On average for Sept-Oct 2015, the magnitude of this bias over the maritime southeast Asian region is 0.17×10^{18} molec cm^{-2} and the corresponding uncertainty is quantified as the standard deviation in the CO total column difference of the 2003-2014 control experiment with respect to the reanalysis itself (~0.14×10^{18} molec cm^{-2}), and scaled with the MOPITT mean CO total column for the current time period (2.3×10^{18} molec cm^{-2}), which is 6%. This is the third C-IFS model uncertainty contribution to the reported 11% total model uncertainty. Note that this uncertainty estimate is here considered independent to the 9% uncertainty due to CO lifetime as discussed above, which however may be correlated to some extent and thus reduce the overall model uncertainty.

The 17% relative uncertainty, see Table 1, attributed to the C-IFS-BG mean bias with respect to MOPITT, is computed as the ratio of the root-mean-square error (RMSE) to the mean excess of 2015 CO columns compared to the CAMS Interim Reanalysis climatology. The RMSE is computed as the time-averaged daily bias, see Fig. 4, left panel, for the bias evolution during Sept-Oct 2015. This is an independent contribution to the uncertainty in the C-IFS-based CO emission constraint, neither related to C-IFS model nor to MOPITT observational uncertainties.

Supplementary Information D.

Using the GFEDv4 Burned Area data (Randerson et al., 2015) here we provide an estimate of the average burn depth of the peatlands. From our total emissions estimate of 692 Tg CO_2 produced in Sept-Oct 2015 over the region, 79% (548 Tg) can be attributed to CO_2 originating from areas with peatland, based on the GFEDv3.1 peatland map. With our emission factor of 1.625 kg CO_2 kg^{-1}DM for peat burning, this implies that 336 Tg DM peat has been lost, which corresponds to 2.1×10^9 m^3 peat volume, assuming an average peat bulk density of 0.16 g cm^{-3} (observed densities range between 0.12-0.20 g cm^{-3} for agricultural and degraded open peatland, Könönen et al., 2015). GFASv4 estimates $2.11\pm1.0\times10^6$ ha were burned during Sept-October over the whole of the Maritime southeast Asia region, of which $0.8\pm0.3\times10^6$ ha can be attributed to peatland. The peat lost by burning is therefore 42 kg Dry Matter per m^2 over the period, resulting in a global average burn depth estimate of 26 cm. The CO_2 emission estimate used in this derivation is based on the optimization procedure employing the MOPITT observations, and is therefore independent of the prior GFAS biomass combustion rate which has been calibrated against GFEDv3.1 and uses a standard burn depth.

For the 1997 situation, GFEDv4 reports a peatland area burnt of $1.7\pm0.9\times10^6$ ha, and associated CO_2 emissions of 2741 Tg × 80% = 2193 Tg CO_2 originating from peat burning. This corresponds to 1349 Tg DM in total, or 78 kg DM per m^2 of burned peatland, equivalent to a global mean 50 cm burn depth and which is broadly in line with Page (2002) and van der Werf et al. (2010).

We refrain from providing an uncertainty budget analysis, but provide a tentative uncertainty estimate of at least 50% caused by uncertainties arising from the GFEDv4 burnt area product and its assumed peat fraction, the estimated CO_2 emissions, its conversion to peat mass, and the assumed peat bulk density.

References

Adler, R.F., G.J. Huffman, A. Chang, R. Ferraro, P. Xie, J. Janowiak, B. Rudolf, U. Schneider, S. Curtis, D. Bolvin, A. Gruber, J. Susskind, and P. Arkin, 2003: The Version 2 Global Precipitation Climatology Project (GPCP) Monthly Precipitation Analysis (1979-Present), J. Hydrometeor., 4,1147-1167.

Akagi, S. K., Yokelson, R. J., Wiedinmyer, C., Alvarado, M. J., Reid, J. S., Karl, T., Crounse, J. D., and Wennberg, P. O.: Emission factors for open and domestic biomass burning for use in atmospheric models, Atmos. Chem. Phys., 11, 4039-4072, doi:10.5194/acp-11-4039-2011, 2011.

Andreae, M. O., and Merlet, P.: Emission of trace gases and aerosols from biomass burning. Global biogeochemical cycles, 15(4), 955-966, 2001.

Deeter, M. N., Worden, H. M., Edwards, D. P., Gille, J. C., and Andrews, A. E.: Evaluation of MOPITT Retrievals of Lower tropospheric Carbon Monoxide over the United States, J. Geophys. Res., 117, D13306, doi:10.1029/2012JD017553, 2012.

Deeter, M. N., S. Martínez-Alonso, D. P. Edwards, L. K. Emmons, J. C. Gille, H. M. Worden, J. V. Pittman, B. C. Daube, and S. C. Wofsy: Validation of MOPITT Version 5 thermal-infrared, near-infrared, and multispectral carbon monoxide profile retrievals for 2000–2011, J. Geophys. Res. Atmos., 118, 6710–6725, doi:10.1002/jgrd.50272, 2013.

Duncan, B. N., I. Bey, M. Chin, L. J. Mickley, T. D. Fairlie, R. V. Martin, and H. Matsueda, Indonesian wildfires of 1997: Impact on tropospheric chemistry, J. Geophys. Res., 108, 4458, doi:10.1029/2002JD003195, D15, 2003.

Emmons, L. K., Walters, S., Hess, P. G., Lamarque, J.-F., Pfister, G. G., Fillmore, D., Granier, C., Guenther, A., Kinnison, D., Laepple, T., Orlando, J., Tie, X., Tyndall, G., Wiedinmyer, C., Baughcum, S. L., and Kloster, S.: Description and evaluation of the Model for Ozone and Related chemical Tracers, version 4 (MOZART-4), Geosci. Model Dev., 3, 43-67, doi:10.5194/gmd-3-43-2010, 2010.

Fisher, J. A., Wilson, S. R., Zeng, G., Williams, J. E., Emmons, L. K., Langenfelds, R. L., Krummel, P. B., and Steele, L. P.: Seasonal changes in the tropospheric carbon monoxide profile over the remote Southern Hemisphere evaluated using multi-model simulations and aircraft observations, Atmos. Chem. Phys., 15, 3217-3239, doi:10.5194/acp-15-3217-2015, 2015.

Flemming, J., Huijnen, V., Arteta, J., Bechtold, P., Beljaars, A., Blechschmidt, A.-M., Diamantakis, M., Engelen, R. J., Gaudel, A., Inness, A., Jones, L., Josse, B., Katragkou, E., Marecal, V., Peuch, V.-H., Richter, A., Schultz, M. G., Stein, O., and Tsikerdekis, A.: Tropospheric chemistry in the Integrated Forecasting System of ECMWF, Geosci. Model Dev., 8, 975-1003, doi:10.5194/gmd-8-975-2015, 2015.

Giglio, L., Descloitres, J., Justice, C. O., & Kaufman, Y. J.: An enhanced contextual fire detection algorithm for MODIS. Remote sensing of environment, 87(2), 273-282, 2003.

Granier, C., B. Bessagnet, T. Bond, A. D'Angiola, H.D.v.d. Gon, G.J. Frost, A. Heil, J.W. Kaiser, S. Kinne, Z. Klimont, S. Kloster, J.-F. Lamarque, C. Liousse, T. Masui, F. Meleux, A. Mieville, T. Ohara, J.-C. Raut, K. Riahi, M.G. Schultz, S.J. Smith, A. Thomson, J.v. Aardenne, G.R.v.d. Werf, and D.P.v. Vuuren: Evolution of anthropogenic and biomass burning emissions of air pollutants at global and regional scales during the 1980-2010 period, Climatic Change, 109(1-2), 163-190, doi:10.1007/s10584-011-0154-1, 2011.

Heil, A., Kaiser, J.W., van derWerf, G. R.,Wooster, M. J., Schultz, M. G., and Dernier van der Gon, H.: Assessment of the Real- Time Fire Emissions (GFASv0) by MACC, Tech. Memo. 628, ECMWF, Reading, UK, 2010.

Ichoku, C. and Kaufman, Y. J.: A method to derive smoke emission rates from MODIS fire radiative energy measurements, IEEE Geosci. Remote. Sci., 43, 2636–2649, 2005.

Inness, A., Blechschmidt, A.-M., Bouarar, I., Chabrillat, S., Crepulja, M., Engelen, R. J., Eskes, H., Flemming, J., Gaudel, A., Hendrick, F., Huijnen, V., Jones, L., Kapsomenakis, J., Katragkou, E., Keppens, A., Langerock, B., de Mazière, M., Melas, D., Parrington, M., Peuch, V. H., Razinger, M., Richter, A., Schultz, M. G., Suttie, M., Thouret, V., Vrekoussis, M., Wagner, A., and Zerefos, C.: Data assimilation of satellite-retrieved ozone, carbon monoxide and nitrogen dioxide with ECMWF's Composition-IFS, Atmos. Chem. Phys., 15, 5275-5303, doi:10.5194/acp-15-5275-2015, 2015a.

Inness, A., Benedetti, A., Flemming, J., Huijnen, V., Kaiser, J. W., Parrington, M., and Remy, S.: The ENSO signal in atmospheric composition fields: emission-driven versus dynamically induced changes, Atmos. Chem. Phys., 15, 9083-9097, doi:10.5194/acp-15-9083-2015, 2015b.

Joyce, R.J., J.E. Janowiak, P. A. Arkin and P. Xie: CMORPH: A Method that Produces Global Precipitation Estimates from Passive Microwave and Infrared Data at High Spatial and Temporal Resolution. J. Hydrometeorology, 5, 487-503, 2004.

Kaiser, J.W., Suttie, M., Flemming, J., Morcrette, J.-J., Boucher, O., and Schultz, M. G: Global real-time fire emission estimates based on space-borne fire radiative power observations. AIP Conf. Proc., 1100:645–648, 2009.

Kaiser, J. W., Heil, A., Andreae, M. O., Benedetti, A., Chubarova, N., Jones, L., Morcrette, J.-J., Razinger, M., Schultz, M. G., Suttie, M., and van der Werf, G. R.: Biomass burning emissions estimated with a global fire assimilation system based on observed fire radiative power, Biogeosciences, 9, 527–554, doi:10.5194/bg-9-527-2012, 2012.

Könönen, M., Jauhiainen, J., Laiho, R., Kusin, K. and Vasander, H.: Physical and chemical properties of tropical peat under stabilised land uses. Mires and Peat 16(8), 2015.

Konovalov, I. B., Berezin, E. V., Ciais, P., Broquet, G., Beekmann, M., Hadji-Lazaro, J., Clerbaux, C., Andreae, M. O., Kaiser, J. W., and Schulze, E.-D.: Constraining CO2 emissions from open biomass burning by satellite observations of co-emitted species: a method and its application to wildfires in Siberia, Atmos. Chem. Phys., 14, 10383-10410, doi:10.5194/acp-14-10383-2014, 2014.

Olivier J., J. Peters, C. Granier, G. Petron, J.F. Muller, and S. Wallens: Present and future surface emissions of atmospheric compounds, POET report #2, EU project EVK2-1999-00011, 2003.

Schneider, U., Becker, A., Finger, P., Meyer-Christoffer, A., Rudolf, B., Ziese, M.: GPCC Full Data Reanalysis Version 7.0 at 0.5°: Monthly Land-Surface Precipitation from Rain-Gauges built on GTS-based and Historic Data. DOI: 10.5676/DWD_GPCC/FD_M_V7_050, 2015.

Schroeder, W., Ellicott, E., Ichoku, C., Ellison, L., Dickinson, M. B., Ottmar, R. D., Clements, C., Hall, D., Ambrosia, V. and Kremens, R.: Integrated active fire retrievals and biomass burning emissions using

complementary near-coincident ground, airborne and spaceborne sensor data. Remote Sensing of Environment, 140:719–730, 2014.

Sindelarova, K., Granier, C., Bouarar, I., Guenther, A., Tilmes, S., Stavrakou, T., Müller, J.-F., Kuhn, U., Stefani, P., and Knorr, W.: Global data set of biogenic VOC emissions calculated by the MEGAN model over the last 30 years, Atmos. Chem. Phys., 14, 9317-9341, doi:10.5194/acp-14-9317-2014, 2014.

van der Werf, G. R., Randerson, J. T., Giglio, L., Collatz, G. J., Mu, M., Kasibhatla, P. S., Morton, D. C., DeFries, R. S., Jin, Y., and van Leeuwen, T. T.: Global fire emissions and the contribution of deforestation, savanna, forest, agricultural, and peat fires (1997–2009), Atmos. Chem. Phys., 10, 11707-11735, doi:10.5194/acp-10-11707-2010, 2010.

Wooster, M. J., Roberts, G., Perry, G. L.W., and Kaufman, Y. J.: Retrieval of biomass combustion rates and totals from fire radiative power observations: FRP derivation and calibration relationships between biomass consumption and fire radiative energy release, J. Geophys. Res., 110, D24311, doi:10.1029/2005JD006318, 2005.

Worden, H. M., Deeter, M. N., Edwards, D. P., Gille, J. C., Drummond, J. R., and Nédélec, P. P.: Observations of near-surface carbon monoxide from space using MOPITT multispectral retrievals, J. Geophys. Res., 115, D18314, doi:10.1029/2010JD014242, 2010.

Supplementary Figures

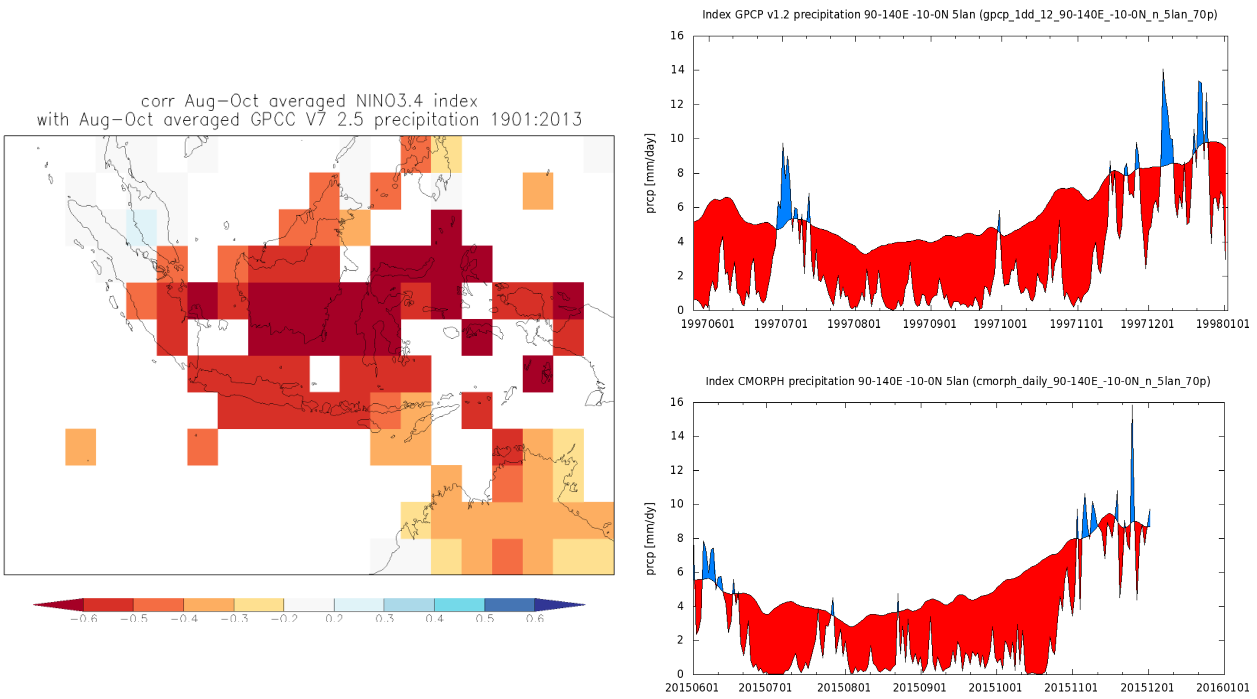

Figure S1. Left: Spatial variation over southeast Asia in temporal correlation between the NINO3.4 Index (measure of the strength of El Niño through sea surface temperature anomalies) and GPCC V7 2.5º precipitation measures (Schneider et al., 2015), calculated for August-October using 1901-2013 historical data. Top right: GPCP 1997 precipitation (Adler et al., 2004) and bottom right: CMORPH 2015 precipitation (Joyce et al., 2004) compared to their respective climatological means (red: deficit, blue excess precipitation), from 1 June to 1 January over land in maritime southeast Asia south of the equator (90°E - 140°E and 10°S - 0°S). The map and timeseries are constructed using the KNMI Climate Explorer, http://climexp.knmi.nl, using gnuplot v4.6 software (http://www.gnuplot.info/), copyright 1986 - 1993, 1998, 2004 Thomas Williams, Colin Kelley.

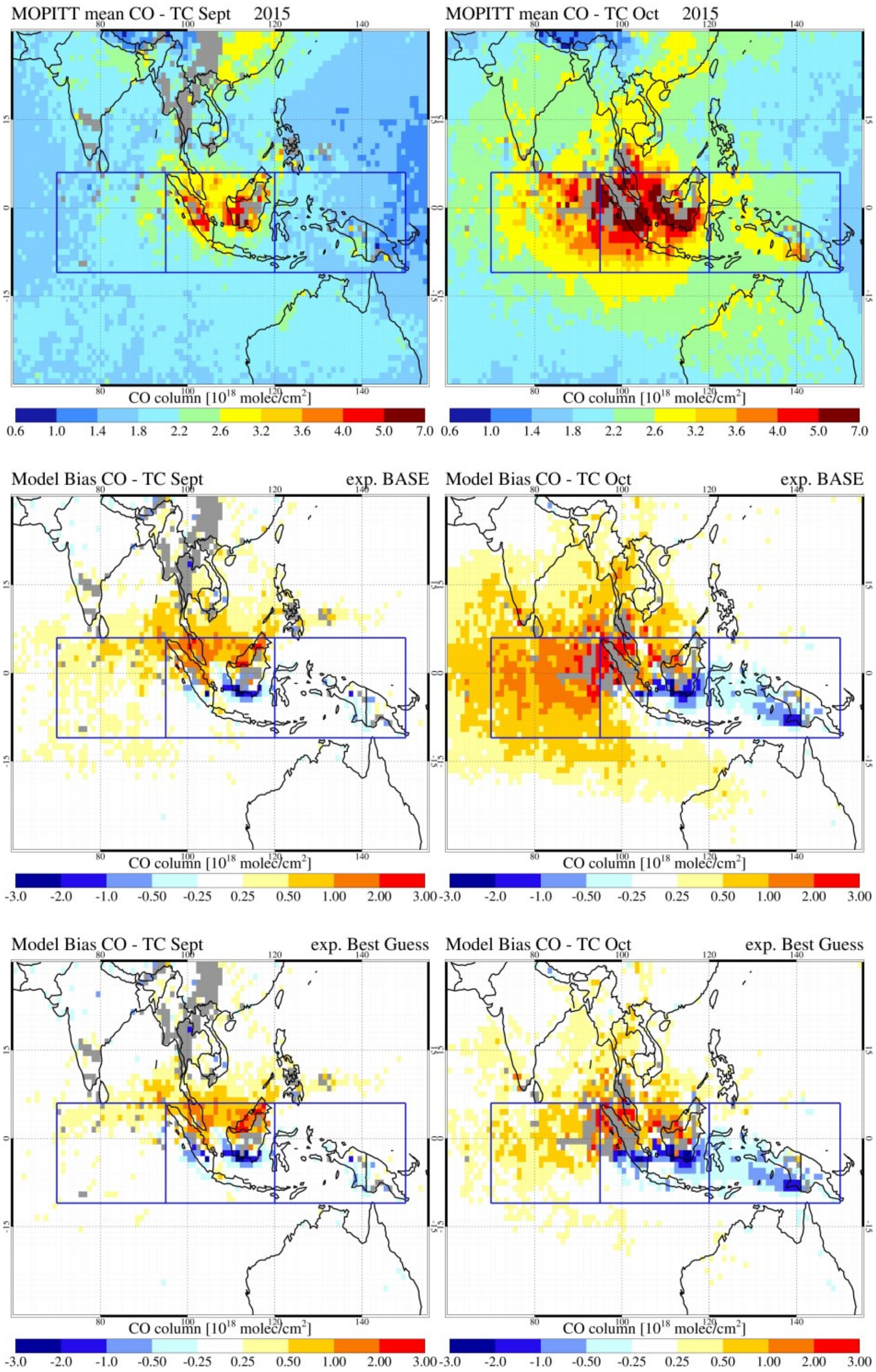

Figure S2. Monthly mean CO total columns of MOPITT-V5T (top) and the bias of the C-IFS GFAS (middle) and C-IFS Best Guess (BG) (bottom) model simulation with respect to MOPITT for September (left) and October (right) 2015. Boxes indicate the regions adopted for the sub-regional evaluations in Fig ED4. The maps were generated using IDL v8.4 software, http://www.exelisvis.com.

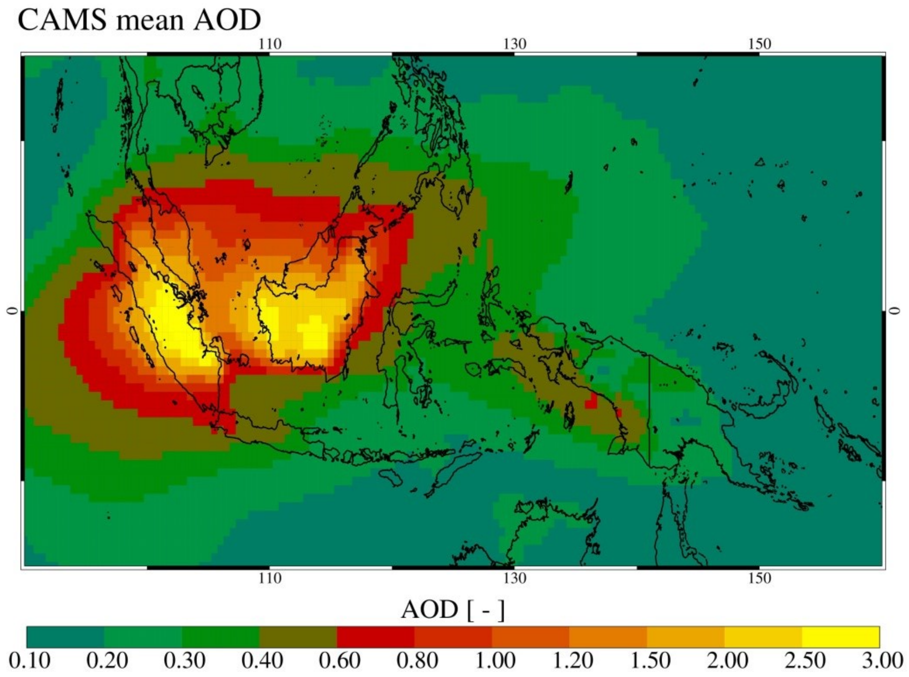

Figure S3. The CAMS operational analysis of the September-October 2015 mean aerosol optical depth (AOD) at 550nm (http://atmosphere.copernicus.eu). The map was generated using IDL v8.4 software, http://www.exelisvis.com.

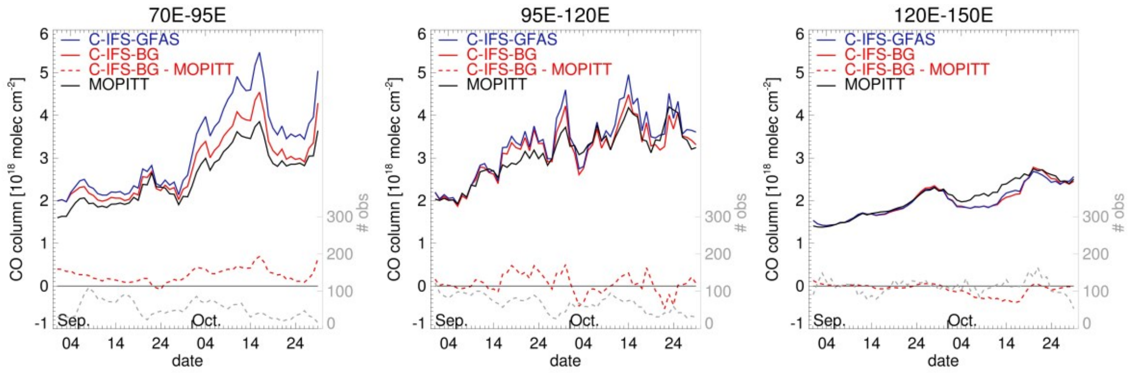

Figure S4: Evolution of C-IFS GFAS (blue) and C-IFS BG (red, solid) 3-day running mean CO columns over three sub-regions as indicated in Figure ED3, compared to MOPITT (black). The bias of CIFS-BG with respect to MOPITT is also given (red, dash). In grey dashes the number of 1°×1° gridded observational samples (right axis).

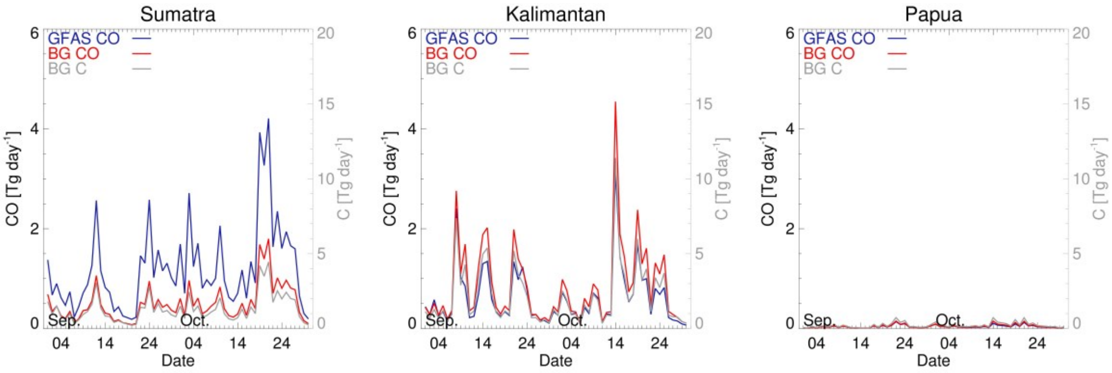

Figure S5: Evolution of GFAS CO emissions (blue) and derived BG CO emissions (red) over Sumatra, Kalimantan, and Papua. In grey the corresponding carbon emissions are shown (right axis).

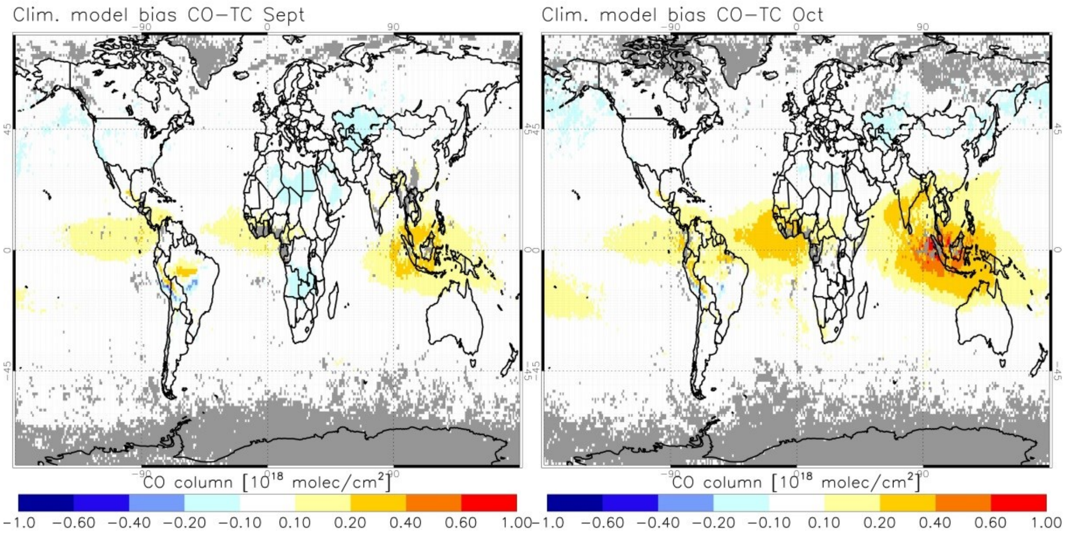

Figure S6. Monthly mean climatological bias in C-IFS computed as the difference in CO total columns between the 2003-2014 control simulation and the corresponding CAMS Interim Reanalysis, for September (left) and October (right). The maps were generated using IDL v8.4 software, http://www.exelisvis.com.
